# Is Bagging Effective in the Classification of Small-Sample Genomic and Proteomic Data?

**DOI:** 10.1155/2009/158368

**Published:** 2009-02-24

**Authors:** TT Vu, UM Braga-Neto

**Affiliations:** 1 Department of Electrical and Computer Engineering, Texas A&M University, College Station, TX 77843-3128, USA

## Abstract

There has been considerable interest recently in the application of bagging in the classification of both gene-expression data and protein-abundance mass spectrometry data. The approach is often justified by the improvement it produces on the performance of unstable, overfitting classification rules under small-sample situations. However, the question of real practical interest is whether the ensemble scheme will improve performance of those classifiers sufficiently to beat the performance of single stable, nonoverfitting classifiers, in the case of small-sample genomic and proteomic data sets. To investigate that question, we conducted a detailed empirical study, using publicly-available data sets from published genomic and proteomic studies. We observed that, under *t*-test and RELIEF filter-based feature selection, bagging generally does a good job of improving the performance of unstable, overfitting classifiers, such as CART decision trees and neural networks, but that improvement was not sufficient to beat the performance of single stable, nonoverfitting classifiers, such as diagonal and plain linear discriminant analysis, or 3-nearest neighbors. Furthermore, as expected, the ensemble method did not improve the performance of these classifiers significantly. Representative experimental results are presented and discussed in this work.

## 1. Introduction

Randomized ensemble methods for classifier design combine the decision of an ensemble of classifiers designed on randomly perturbed versions of the available data [1–5]. The combination is often done by means of majority voting among the individual classifier decisions [4–6], whereas the data perturbation usually employs the bootstrap resampling approach, which corresponds to sampling uniformly with replacement from the original data [7,8]. The combination of bootstrap resampling and majority voting is known as bootstrap aggregation or *bagging* [4,5].

There has been considerable interest recently in the application of bagging in the classification of both gene-expression data [9–12] and protein-abundance mass spectrometry data [13–18]. However, there is scant theoretical justification for the use of this heuristic, other than the expectation that combining the decision of several classifiers will regularize and improve the performance of unstable overfitting classification rules, such as unpruned decision trees, provided one uses a large enough number of classifiers in the ensemble [4,5]. It is also claimed that ensemble rules "do not overfit," meaning that classification error converges as the number of component classifiers tends to infinity [5].

However, the main performance issue is not whether the ensemble scheme improves the classification error of a single unstable overfitting classifier, or whether its classification error converges to a fixed limit; these are important questions, which have been studied in the literature (in particular when the component classifiers are decision trees) [5,19–23], but the question of main practical interest is whether the ensemble scheme will improve the performance of unstable overfitting classifiers *sufficiently* to beat the performance of single stable, nonoverfitting classifiers, particularly in small-sample settings. Therefore, there is a pressing need to examine rigorously the suitability and validity of the ensemble approach in the classification of small-sample genomic and proteomic data. In this paper, we present results from a comprehensive empirical study concerning the effect of bagging on the performance of several classification rules, including diagonal and plain linear discriminant analysis, 3-nearest neighbors, CART decision trees, and neural networks, using real data from published microarray and mass spectrometry studies. Here we are concerned exclusively with the performance in terms of the true classification error, and therefore we employ filter-based feature selection and holdout estimation based on large samples in order to allow accurate classification error estimation. Similar studies recently published [11,12] rely on small-sample wrapper feature selection and small-sample error estimation methods, which will obscure the issue of how bagging really affects the true classification error. In particular, there is evidence that filter-based feature selection outperforms wrapper feature selection in small-sample settings [24]. In our experiments, we employ the one-tailed paired *t*-test to assess whether the expected true classification error is significantly smaller for the bagged classifier as opposed to the original base classifier, under different number of samples, dimensionality, and number of classifiers in the ensemble. Clearly, the heuristic is beneficial for the particular classification rule if and only there is a significant decrease in expected classification error, otherwise the procedure is to be avoided; however the magnitude of improvement is also a factor—a small improvement in performance may not be worth the extra computation required (which is roughly  times larger for the bagging classifier, where  is the number of classifiers in the ensemble). The full results of the empirical study are available on a companion website http://www.ece.tamu.edu/~ulisses/bagging/index.html .

## 2. Randomized Ensemble Classification Rules

Classification involves a *feature vector* in a feature space , a *label*, and a *classifier*, such that  attempts to predict the value of  for a given observation . The joint *feature-label distribution* of the pair  completely characterizes the stochastic properties of the classification problem. In practice, a classification rule is used to design a classifier based on sample training data. Working formally, a *classification rule* is a mapping , which takes an i.i.d. sample  of feature-label pairs drawn from the feature-label distribution to a *designed classifier*. The *classification error* is the probability that classification is erroneous given the sample data, that is, . Note that the classification error is random only through the training data . The *expected classification error* is the average classification error over all possible sample data sets; it is a fixed parameter of the classification rule and feature-label distribution, and used as the measure of performance of the former given the latter.

Randomization approaches based on resampling can be seen as drawing i.i.d. samples  from a surrogate joint-feature label distribution , which is a function of the original training data . In the bootstrap resampling approach, one has , and the randomized sample  corresponds to sampling uniformly  training points from *with* replacement. This corresponds to using the *empirical distribution* of the data  as the surrogate joint-feature label distribution ; the empirical distribution assigns discrete probability mass  at each observed data point in . Some of the original training points may appear multiple times, whereas others may not appear at all in the *bootstrap sample*. Note that, given , the bootstrap sample  is conditionally independent from the original feature-label distribution .

In aggregation by majority voting, a classifier is obtained based on majority voting among individual classifiers designed on the randomized samples  using the original classification rule . This leads to an *ensemble classification rule*, such that(1)

for , where expectation is with respect to the random mechanism , fixed at the observed value of . For bootstrap majority voting, or bagging, the expectation in (1) usually has to be approximated by Monte Carlo sampling, which leads to the "bagged" classifier:(2)

where the classifiers  are designed by the original classification rule  on bootstrap samples , for , for large enough  (notice the parallel with the development in [25], particulary equations (2.8)–(2.10), and accompanying discussion).

The issue of how large  has to be so that (2) is a good Monte Carlo approximation is a critical issue in the application of bagging. Note that  represents the number of classifiers that must be designed to be part of the ensemble, so that a computational problem may emerge if  is made too large. In addition, even if a suitable  is found, the performance of the ensemble must be compared to that of the base classification rule, to see if there is significant improvement. Even more importantly, the performance of the ensemble has to be compared to that of other classification rules; that the ensemble improves the performance of an unstable overfitting classifier is of small value if it can be bested by a single stable, nonoverfitting classifier. In the next section, we present a comprehensive empirical study that addresses these questions.

## 3. Experimental Study

In this section, we report the results obtained from a large simulation study based on publicly-available patient data from genomic and proteomic studies, which measured the performance of the bagging heuristic through the expected classification error, for varying number of component classifiers, sample size, and dimensionality.

### 3.1. Methods

We considered in our experiment several classification rules, listed here in order of complexity: diagonal linear discriminant analysis (DLDA), linear discriminant analysis (LDA), 3-nearest neighbors (3NN), decision trees (CART), and neural networks (NNET) [26,27]. DLDA is an extension of LDA where only the diagonal elements (the variances) of the covariance matrix are estimated, while the off-diagonal elements (the covariances) are assumed to be zero. Bagging is applied to each of these base classification rules and its performance recorded for varying number of individual classifiers. The neural network consists of a one-hidden layer with 4 nodes and standard sigmoids as nonlinearities. The network is trained by Levenberg-Marquardt optimization with a maximum of 30 iterations. CART is applied with a stopping criterion. Splitting is stopped when there are fewer than 3 points in a given node. This is distinct from the approach advocated in [5] for random forests, where unpruned, fully grown trees are used instead; the reason for this is that we did not attempt to implement the approach in [5] (which involves concepts as random node splitting and is thus specific to decision trees), but rather to study the behavior of bagging, which is the centerpiece of such ensemble methods, across different classification rules. Resampling is done by means of *balanced* bootstrapping, where all samples are made to appear exactly the same number of times in the computation [28].

We selected data sets with large number  of samples (see below) in order to be able to estimate the true error accurately using held out testing data. In each case, 1000 training data sets of size  were drawn uniformly and independently from the total pool of  samples. The training data are drawn in a stratified fashion, following the approximate proportion of each class in the original data. Based on the training data, a filter-based gene selection step is employed to select the top  discriminating genes; we considered in this study . The univariate feature selection methods used in the filter step are the Welch two-sample *t*-test [29] and the RELIEF method [30]—in the latter case, we employ the 1-nearest neighbor method when searching for hits and misses. After classifier design, the true classification error for each data set of size  is approximated by a holdout estimator, whereby the  sample points not drawn are used as the test set (a good approximation to the classification error, given that ). The expected classification error is then estimated as the sample mean of classification error over the 1000 training data sets. The sample size  is kept small, as we are interested in the small-sample properties of bagging. Note also that we also must have  in order to provide for large enough testing sets, as well as to make sure that consecutive training sets do not significantly overlap, so that the expected classification error can be accurately approximated. As can be easily verified, the expected ratio of overlapping sample points between two samples of size  from a population of size  is given simply by . In all cases considered here the expected overlap is around 20% less, which we consider to be acceptable, except in the case of the lung cancer data set with . This latter case is therefore not included in our results. The one-tailed paired *t*-test is employed to assess whether the ensemble classifier has an expected error that is significantly smaller than that of the corresponding individual classifier.

### 3.2. Data Sets

We utilized the following publicly-available data sets from published studies in order to study the performance of bagging in the context of genomics and proteomics applications.

#### 3.2.1. Breast Cancer Gene Expression Data

These data come from the breast cancer classification study in [31], which analyzed  gene-expression microarrays containing a total of 25760 transcripts each. Filter-based feature selection was performed on a 70-gene prognosis profile, previously published by the same authors in [32]. Classification is between the good-prognosis class (115 samples), and the poor-prognosis class (180 samples), where prognosis is determined retrospectively in terms of survivability [31].

#### 3.2.2. Lung Cancer Gene Expression Data

We employed here the data set "A" from the study in [33] on nonsmall-cell lung carcinomas (NSCLC), which analyzed  gene-expression microarrays containing a total of 12600 transcripts each. NSCLC is subclassified as adenocarcinomas, squamous cell carcinomas and large-cell carcinomas, of which adenocarcinomas are the most common subtypes and of interest to classify from other subtypes of NSCLC. Classification is thus between adenocarcinomas (139 samples) and non-adenocarcinomas (47 samples).

#### 3.2.3. Prostate Cancer Protein Abundance Data

Given the recent keen interest on deriving serum-based proteomic biomarkers for the diagnosis of cancer [34], we also included in this study data from a proteomic study of prostate cancer reported in [35]. It consists of SELDI-TOF mass spectrometry of  samples, which yield mass spectra for 45000 m/z (mass over charge) values. Filter-based feature selection is employed to find the top discriminatory m/z values to be used in the experiment. Classification is between prostate cancer patients (167 samples) and noncancer patients, including benign prostatic hyperplasia and healthy patients (159 samples). We use the raw spectra values, without baseline subtraction, as we found that this leads to better classification rates.

### 3.3. Results and Discussion

We present results for sample sizes  and  and dimensionality  and , which are representative of the full set of results, available on the companion websitehttp://www.ece.tamu.edu/~ulisses/bagging/index.html. The case  is displayed in Tables 1, 2, and 3, each of which corresponds to a different data set. Each table displays the expected classification error as a function of the number  of classifiers used in the ensemble, for different base classification rules, feature selection methods, and sample sizes. We used in all cases an odd number  of classifiers in the ensembles, to avoid tie-breaking issues. Errors that are smaller for the ensemble classifier as compared to a single classifier at a 99% significance level, according to the one-tailed paired *t*-test, are indicated by bold-face type. This allows one to immediately observe that bagging is able to improve the performance of the unstable overfitting CART and NNET classifiers; in most cases, a small ensemble is required, and the improvement in performance is substantial. In contrast, bagging does not improve the performance of the stable, nonoverfitting DLDA, LDA, and 3NN classifiers, except via a large ensemble; and even so the improvement in magnitude is quite small, and certainly does not justify the extra computational cost (note that in the case of the simplest classification rule, DLDA, there is no improvement at all). This is in agreement with what is known about the ensemble approach (e.g., see [5]).

**Table 1:** Expected classification error of selected experiments for breast cancer gene-expression data under two different features selection methods (*t*-test and RELIEF) for .

Rule	FS		Single										
DLDA	*t*-test	20	0.202	0.215	0.208	0.206	0.204	0.204	0.204	0.204	0.203	0.204	0.203
DLDA	*t*-test	40	0.198	0.205	0.201	0.200	0.200	0.199	0.199	0.199	0.199	0.199	0.199
DLDA	RELIEF	20	0.202	0.215	0.207	0.206	0.204	0.204	0.204	0.203	0.203	0.203	0.203
DLDA	RELIEF	40	0.198	0.206	0.201	0.201	0.200	0.200	0.199	0.199	0.199	0.199	0.199

LDA	*t*-test	20	0.212	0.237	0.224	0.220	0.217	0.217	0.216	0.216	0.215	0.215	0.214
LDA	*t*-test	40	0.204	0.217	0.209	0.208	0.207	0.206	0.206	0.206	0.205	0.205	0.205
LDA	RELIEF	20	0.213	0.239	0.225	0.222	0.219	0.218	0.218	0.217	0.216	0.216	0.216
LDA	RELIEF	40	0.203	0.218	0.210	0.207	0.206	0.206	0.205	0.205	0.205	0.205	0.205

3NN	*t*-test	20	0.230	0.281	0.246	0.241	0.235	0.234	0.231	0.231	0.230	0.229	**0.229**
3NN	*t*-test	40	0.228	0.274	0.241	0.235	0.231	0.229	0.228	0.227	**0.226**	**0.226**	**0.225**
3NN	RELIEF	20	0.234	0.282	0.248	0.242	0.238	0.236	0.234	0.234	0.233	**0.233**	**0.232**
3NN	RELIEF	40	0.227	0.271	0.241	0.235	0.231	0.229	0.227	0.227	**0.226**	**0.225**	**0.225**

CART	*t*-test	20	0.259	0.297	0.263	0.256	**0.250**	**0.247**	**0.246**	**0.244**	**0.243**	**0.242**	**0.242**
CART	*t*-test	40	0.257	0.294	0.258	**0.252**	**0.245**	**0.244**	**0.242**	**0.240**	**0.239**	**0.239**	**0.237**
CART	RELIEF	20	0.263	0.299	0.265	**0.258**	**0.253**	**0.250**	**0.247**	**0.247**	**0.245**	**0.245**	**0.244**
CART	RELIEF	40	0.256	0.293	0.260	**0.253**	**0.245**	**0.244**	**0.241**	**0.240**	**0.239**	**0.239**	**0.238**

NNET	*t*-test	20	0.252	0.293	**0.246**	**0.240**	**0.230**	**0.230**	**0.225**	**0.224**	**0.223**	**0.222**	**0.221**
NNET	*t*-test	40	0.226	0.256	0.225	**0.219**	**0.215**	**0.213**	**0.212**	**0.210**	**0.210**	**0.209**	**0.209**
NNET	RELIEF	20	0.255	0.298	**0.248**	**0.240**	**0.233**	**0.232**	**0.229**	**0.228**	**0.226**	**0.225**	**0.224**
NNET	RELIEF	40	0.230	0.260	0.227	**0.220**	**0.216**	**0.213**	**0.213**	**0.212**	**0.211**	**0.210**	**0.209**

Bold-face type indicates the values that are smaller for the ensemble classifier as compared to a single classifier at a 99% significance level, according to the one-tailed paired *t*-test.

**Table 2:** Expected classification error of selected experiments for lung cancer gene-expression data under two different features selection methods (*t*-test and RELIEF) for .

Rule	FS		Single										
DLDA	*t*-test	20	0.190	0.191	0.190	0.190	**0.189**	**0.190**	**0.189**	**0.189**	0.190	**0.190**	**0.190**
DLDA	*t*-test	40	0.186	0.187	0.186	0.186	**0.186**	0.186	0.186	**0.186**	**0.186**	0.186	0.186
DLDA	RELIEF	20	0.235	0.253	0.238	0.239	0.235	0.236	**0.233**	0.233	0.234	**0.232**	**0.233**
DLDA	RELIEF	40	0.207	0.212	0.209	0.208	0.207	0.207	0.207	0.207	0.207	0.207	0.206

LDA	*t*-test	20	0.201	0.206	0.203	0.203	0.202	0.202	0.203	0.202	0.202	0.202	0.203
LDA	*t*-test	40	0.192	0.194	0.193	0.193	0.193	0.193	0.192	0.192	0.193	0.192	0.192
LDA	RELIEF	20	0.262	0.295	0.274	0.271	0.265	0.265	0.263	0.263	0.260	0.261	0.261
LDA	RELIEF	40	0.208	0.223	0.214	0.213	0.212	0.212	0.210	0.211	0.210	0.210	0.208

3NN	*t*-test	20	0.122	0.151	0.130	0.126	0.124	0.123	0.122	0.121	0.121	0.121	**0.120**
3NN	*t*-test	40	0.123	0.147	0.129	0.127	0.125	0.124	0.123	0.123	**0.122**	**0.122**	**0.121**
3NN	RELIEF	20	0.247	0.334	0.265	0.258	0.249	0.248	0.246	0.247	**0.244**	**0.244**	**0.243**
3NN	RELIEF	40	0.232	0.317	0.252	0.243	0.238	0.235	0.234	0.233	0.232	**0.231**	**0.230**

CART	*t*-test	20	0.160	0.182	0.161	**0.155**	**0.152**	**0.151**	**0.150**	**0.149**	**0.148**	**0.148**	**0.147**
CART	*t*-test	40	0.156	0.177	0.155	**0.150**	**0.146**	**0.145**	**0.144**	**0.143**	**0.142**	**0.142**	**0.142**
CART	RELIEF	20	0.297	0.302	**0.280**	**0.274**	**0.269**	**0.267**	**0.266**	**0.264**	**0.263**	**0.262**	**0.263**
CART	RELIEF	40	0.297	0.297	**0.273**	**0.268**	**0.263**	**0.261**	**0.260**	**0.258**	**0.257**	**0.257**	**0.256**

NNET	*t*-test	20	0.216	0.244	0.235	0.232	0.231	0.229	0.228	0.228	0.227	0.227	0.226
NNET	*t*-test	40	0.195	0.232	0.215	0.212	0.208	0.207	0.205	0.204	0.203	0.202	0.202
NNET	RELIEF	20	0.239	0.257	0.247	0.247	0.244	0.242	0.242	0.241	0.242	0.242	0.241
NNET	RELIEF	40	0.231	0.252	0.242	0.241	0.238	0.236	0.235	0.234	0.234	0.235	0.233

Bold-face type indicates the values that are smaller for the ensemble classifier as compared to a single classifier at a 99% significance level, according to the one-tailed paired *t*-test.

**Table 3:** Expected classification error of selected experiments for prostate cancer protein-abundance data under two different features selection methods (*t*-test and RELIEF) for *p* = 2.

Rule	FS		Single										
DLDA	*t*-test	20	0.188	0.211	0.199	0.196	0.194	0.194	0.193	0.192	0.191	0.191	0.191
DLDA	*t*-test	40	0.187	0.207	0.196	0.194	0.192	0.191	0.191	0.191	0.190	0.190	0.189
DLDA	RELIEF	20	0.468	0.523	0.492	0.484	0.477	0.475	0.471	0.472	0.469	0.467	0.466
DLDA	RELIEF	40	0.458	0.502	0.477	0.474	0.465	0.469	0.465	0.463	0.462	0.463	0.460

LDA	*t*-test	20	0.212	0.241	0.225	0.222	0.219	0.218	0.216	0.216	0.215	0.215	0.215
LDA	*t*-test	40	0.198	0.224	0.210	0.208	0.205	0.204	0.203	0.202	0.202	0.202	0.201
LDA	RELIEF	20	0.422	0.492	0.449	0.435	0.426	0.426	0.422	0.419	0.417	**0.415**	**0.410**
LDA	RELIEF	40	0.416	0.479	0.440	0.433	0.426	0.421	0.420	0.418	0.416	0.415	0.413

3NN	*t*-test	20	0.187	0.251	0.203	0.195	0.192	0.189	0.187	0.187	0.186	**0.185**	**0.185**
3NN	*t*-test	40	0.153	0.208	0.168	0.162	0.158	0.156	0.154	0.153	**0.152**	**0.152**	**0.151**
3NN	RELIEF	20	0.268	0.355	0.307	0.299	0.287	0.284	0.280	0.278	0.277	0.276	0.275
3NN	RELIEF	40	0.222	0.283	0.248	0.239	0.233	0.231	0.229	0.228	0.226	0.226	0.224

CART	*t*-test	20	0.232	0.247	**0.223**	**0.218**	**0.213**	**0.210**	**0.209**	**0.209**	**0.208**	**0.209**	**0.208**
CART	*t*-test	40	0.213	0.219	**0.198**	**0.194**	**0.189**	**0.189**	**0.187**	**0.185**	**0.185**	**0.185**	**0.184**
CART	RELIEF	20	0.244	0.284	0.259	0.256	0.251	0.249	0.247	0.245	0.244	0.244	0.243
CART	RELIEF	40	0.222	0.250	0.233	0.229	0.226	0.225	0.224	0.223	0.223	0.223	0.221

NNET	*t*-test	20	0.297	0.300	**0.271**	**0.266**	**0.260**	**0.259**	**0.256**	**0.256**	**0.254**	**0.254**	**0.253**
NNET	*t*-test	40	0.277	0.274	**0.254**	**0.248**	**0.244**	**0.244**	**0.240**	**0.241**	**0.239**	**0.239**	**0.239**
NNET	RELIEF	20	0.345	0.382	**0.337**	**0.324**	**0.318**	**0.314**	**0.313**	**0.313**	**0.312**	**0.309**	**0.307**
NNET	RELIEF	40	0.329	0.348	**0.312**	**0.303**	**0.295**	**0.294**	**0.290**	**0.290**	**0.290**	**0.288**	**0.289**

Bold-face type indicates the values that are smaller for the ensemble classifier as compared to a single classifier at a 99% significance level, according to the one-tailed paired *t*-test.

**Figure 1:**
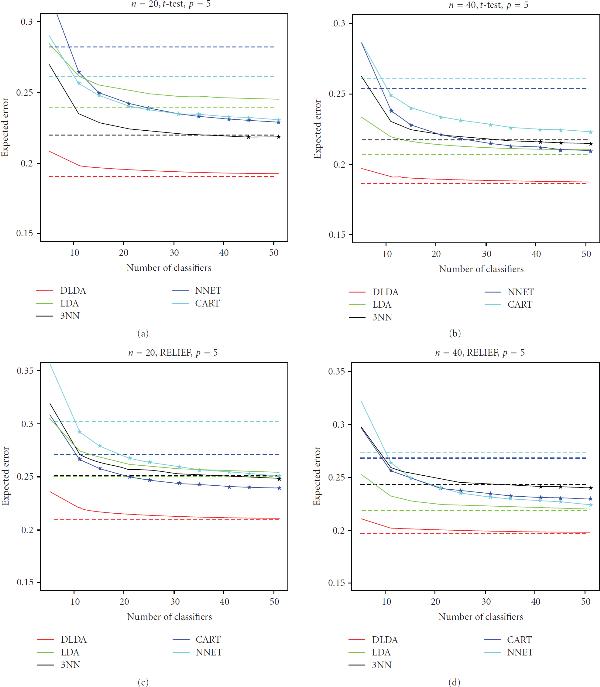
**Expected classification error as a function of number of classifiers in the ensemble for selected experiments with the breast cancer gene expression data (full results available on the companion website)**. The error of a single classifier is indicated by a horizontal dashed line. Marks indicate the values that are smaller for the ensemble classifier as compared to a single classifier at a 99% significance level, according to the one-tailed paired *t*-test.

**Figure 2:**
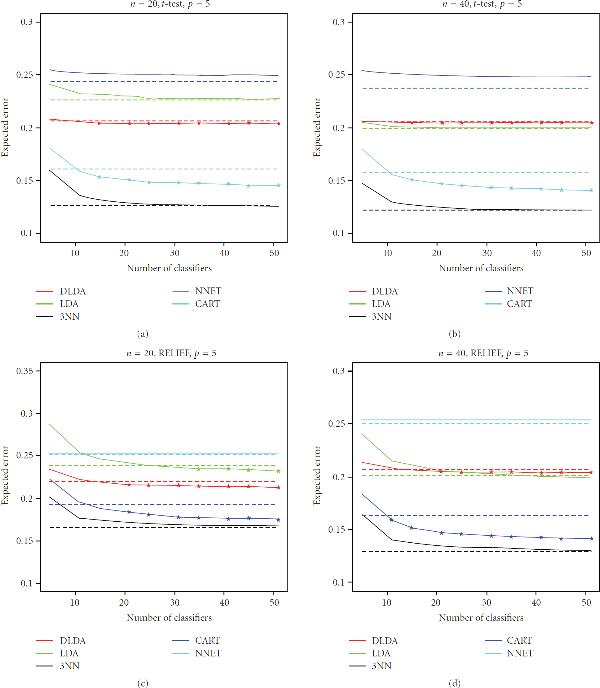
**Expected classification error as a function of number of classifiers in the ensemble for selected experiments with the lung cancer gene expression data (full results available on the companion website)**. The error of a single classifier is indicated by a horizontal dashed line. Marks indicate the values that are smaller for the ensemble classifier as compared to a single classifier at a 99% significance level, according to the one-tailed paired *t*-test.

**Figure 3:**
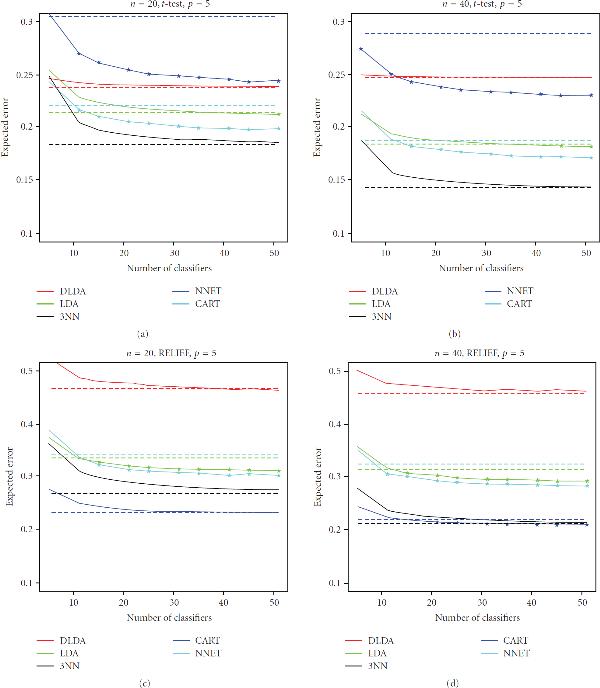
**Expected classification error as a function of number of classifiers in the ensemble for selected experiments with the prostate cancer protein abundance data (full results available on the companion website)**. The error of a single classifier is indicated by a horizontal dashed line. Marks indicate the values that are smaller for the ensemble classifier as compared to a single classifier at a 99% significance level, according to the one-tailed paired *t*-test.

However, of larger interest here is the performance of the ensemble against a single instance of the stable, nonoverfitting classifiers. This can be better visualized in the plots of Figures 1, 2, and 3, which display the expected classification errors as a function of number of component classifiers in the ensemble, for the case . The error of a single classifier is indicated by a horizontal dashed line. Marks indicate the values that are smaller for the ensemble classifier as compared to a single component classifier at a 99% significance level, according to the one-tailed paired *t*-test. One observes that as ensemble size increases, classification error decreases and tends to converge to a fixed value (in agreement with [5]), but we can also see that the error is usually larger at very small ensemble sizes, as compared to the error of the individual classifier. We can again observe that, in most cases, bagging is able to improve the performance of CART and NNET, but that is not significantly so, or at all, for DLDA, LDA, and 3NN. More importantly, we can see that the improvement on the performance of CART and NNET is not sufficient to beat the performance of single DLDA, LDA, or 3NN classifiers (with the exception of the prostate cancer data with RELIEF feature selection, which we comment on below).

As we can see in Figures 1–3, the breast cancer gene-expression data produces linear features that favor single DLDA and LDA classifiers (the latter do not perform so well at , due to the difficulty of estimating the entire covariance matrix at this sample size, which affects DLDA less), while the lung cancer gene-expression data produce nonlinear features, in which case, according to the results, the best option overall is to use a single 3NN classifier, followed closely by a bagged NNET in *t*-test feature selection and a bagged CART in RELIEF feature selection. The case of the prostate cancer proteomic data is peculiar in that it presents the only case where the best option was not a DLDA, LDA, or 3NN classifier, but in fact a single CART classifier, namely, the case  (with either  or ) for RELIEF feature selection (the results for *t*-test feature selection, on the other hand, are very similar to the ones obtained for the lung cancer data set). Note that, in this case, the best performance is achieved by a single CART classifier, rather than the ensemble CART scheme. We also point out that the classification errors obtained with *t*-test feature selection are smaller than the ones obtained with RELIEF feature selection, indicating that RELIEF is not a good option in this case due to the very small-sample size (in fact, there is evidence that *t*-test filter-based feature selection may be the method of choice in small-sample cases [24]), in the case , the difference between 3NN and CART essentially disappears. It is also interesting that in the case  and , for RELIEF feature selection, bagging is able to improve the performance of LDA by a good margin in the case of the prostate cancer data. This is due to the fact that the combination of LDA and RELIEF feature selection produce an unstable overfitting classification rule at this acute small-sample scenario.

The results obtained with *t*-test feature selection are consistent across all data sets. When using RELIEF feature selection, there is a degree of contrast between the results for the prostate cancer protein-abundance data set and the ones for the gene-expression data sets, which may be attributed to the differences in technology as well as the fact that we do not employ baseline subtraction for the proteomics data in order to achieve better classification rates.

We remark that results are not expected to change much if ensemble sizes are increased further (beyond ), as can be seen from convergence of the expected classification error curves in Figures 1–3.

## 4. Conclusion

In this paper we conducted a detailed empirical study of the ensemble approach to classification of small-sample genomic and proteomic data. The main performance issue is not whether the ensemble scheme improves the classification error of an unstable overfitting classifier (e.g., CART, NNET), or whether its classification error converges to a fixed limit; but rather whether the ensemble scheme will improve performance of the unstable overfitting classifier *sufficiently* to beat the performance of single stable, nonoverfitting classifiers (e.g., DLDA, LDA, and 3NN). We observed that this never was the case for any of the data sets and experimental conditions considered here, except in the case of the proteomics data set with RELIEF feature selection in acute small-sample cases, when nevertheless the performance of a single unstable overfitting classifier (in this case, CART) was better or comparable to the corresponding ensemble classifier. We observed that in most cases bagging does a good (sometimes, admirable) job of improving the performance of unstable overfitting classifiers, but that improvement was not enough to beat the performance of single stable nonoverfitting classifiers.

The main message to be gleaned from this study by practitioners is that the use of bagging in classification of small-sample genomics and proteomics data increases computational cost, but is not likely to improve overall classification accuracy over other, more simple, approaches. The solution we recommend is to use simple classification rules and avoid bagging in these scenarios. It is important to stress that we do not give a definitive recommendation on the use of the random forest method for small-sample genomics and proteomics data; however, we do think that this study does provide a step in that direction, since the random forest method depends partly, if not significantly, for its success on the effectiveness of bagging. Further research is needed to investigate this question.
